# Identification of a Novel Chitinase from *Bacillus paralicheniformis*: Gene Mining, Sequence Analysis, and Enzymatic Characterization

**DOI:** 10.3390/foods13111777

**Published:** 2024-06-05

**Authors:** Xianwen Ma, Dian Zou, Anying Ji, Cong Jiang, Ziyue Zhao, Xiaoqi Ding, Zongchen Han, Pengfei Bao, Kang Chen, Aimin Ma, Xuetuan Wei

**Affiliations:** State Key Laboratory of Agricultural Microbiology, College of Food Science and Technology, Huazhong Agricultural University, Wuhan 430070, China; xianwenma@webmail.hzau.edu.cn (X.M.); dianzou@mail.hzau.edu.cn (D.Z.); anyingji@webmail.hzau.edu.cn (A.J.); jiangcong409@163.com (C.J.); zhaoziyue@webmail.hzau.edu.cn (Z.Z.); xiaoqiding0723@163.com (X.D.); hanzongchen1997@163.com (Z.H.); baopengfei@webmail.hzau.edu.cn (P.B.); cingk123@163.com (K.C.); aiminma@mail.hzau.edu.cn (A.M.)

**Keywords:** chitinase, *Bacillus paralicheniformis*, gene *ch1*, recombinant expression, enzymatic characterization

## Abstract

In this study, a novel strain for degrading chitin was identified as *Bacillus paralicheniformis* HL37, and the key chitinase CH1 was firstly mined through recombinant expression in *Bacillus amyloliquefaciens* HZ12. Subsequently, the sequence composition and catalytic mechanism of CH1 protein were analyzed. The molecular docking indicated that the triplet of Asp526, Asp528, and Glu530 was a catalytic active center. The enzymatic properties analysis revealed that the optimal reaction temperature and pH was 65 °C and 6.0, respectively. Especially, the chitinase activity showed no significant change below 55 °C and it could maintain over 60% activity after exposure to 85 °C for 30 min. Moreover, the optimal host strain and signal peptide were obtained to enhance the expression of chitinase CH1 significantly. As far as we know, it was the first time finding the highly efficient chitin-degrading enzymes in *B. paralicheniformis*, and detailed explanations were provided on the catalytic mechanism and enzymatic properties on CH1.

## 1. Introduction

Chitin is alkaline polysaccharide extracted from the shells of marine crustaceans, and it is the second most abundant biopolymer resource in the world [1]. Chitin is widely used as an industrial clothing fabric [2], feed supplements [3], and a wound healing agent in medicine [4]. Chitin can be hydrolyzed to produce N-acetylglucosamine (GlcNAc) and (GlcNAc)_2_, and they have multiple biological activities, such as antibacterial, antioxidant, and immunostimulatory activities [5,6,7,8]. At present, GlcNAc and (GlcNAc)_2_ have extensive applications in fields, such as food, skincare, and biomedicine skincare [9,10,11,12,13]. In the food industry, GlcNAc is used as a dietary supplement [9]. In the field of biomedicine, GlcNAc can alleviate symptoms of osteoarthritis [14]. GlcNAc exhibits a chondroprotective action by inhibiting type II collagen degradation in the articular cartilage [14]. Especially, GlcNAc-related products are very popular in European, American, and Japanese markets, and they are mainly used as a dietary supplement for the prevention and treatment of osteoarthritis [15,16,17]. In the skincare industry, GlcNAc can inhibit melanin production and reduce the appearance of wrinkles [10,11]. (GlcNAc)_2_ can be used as the inducer of bacterial chitinase [12], anti-diabetes activity, lipid-lowering activity, and antioxidant properties [13].

At present, the methods for degradation of chitin mainly include acid hydrolysis and biodegradation. Among them, the acid hydrolysis method has low cost but it uses a large amount of acid and alkali during the preparation process, which seriously pollutes the environment [18,19]. Biodegradation methods include microbial fermentation and enzymatic methods, and the essence is that chitinase plays a degrading role. The fermentation method utilizes chitinase produced by microorganisms to degrade chitin, and many microorganisms can directly produce GlcNAc and (GlcNAc)_2_ through fermentation of chitin, including *Escherichia coli*, *Bacillus subtilis*, *Corynebacterium glutamicum*, and *Aspergillus niger* [20,21,22,23]. The enzymatic method utilizes extracted chitinase to degrade chitin into GlcNAc or (GlcNAc)_2_, and chitinase directly reacts with substrate chitin rapidly [5,24]. Both methods have the advantages of low cost, gentle reaction, and high safety [25,26], but the bio enzymes produced by fermentation of wild strains have low activity and are unresistant to high temperatures. Therefore, it has great industrial prospects for mining efficient chitinase by the biodegradation method.

Previous studies have reported a wide variety of species with the capacity to manufacture chitinase, including *Serratia marcescens* [27], *Trichoderma harzianum* [28], *Bacillus aryabhattai* [29], *Aeromonas caviae* [30], *Paenibacillus barengoltzii* [23], *B. subtilis* [31], and *Cellulosimicrobium funkei* [32]. Many studies have shown that it is feasible to express heterologous chitinase in type strains for biodegrading chitin, such as *E. coli*, *B. subtilis*, and *Pichia pastoris* [30,33,34]. However, the expression system of *E. coli* produces endotoxins, seriously threatening the safety of food and drugs [35]. The commonly used yeast is eukaryotes, with complex operations and a long fermentation cycle [36]. Consequently, as many *Bacillus* spp. are food-grade safe strains and have a strong ability to produce extracellular enzymes and grow rapidly, *Bacillus* enzyme preparations have found extensive application in the food business [37,38]. Therefore, *Bacillus* species have the potential to express the chitinases. 

In this study, the *Bacillus paralicheniformis* HL37 with high degradation ability of chitin was obtained through thermostability screening, and the key enzyme chitinase CH1 was identified by recombinant expression and sequence analysis. Subsequently, we investigated the specific degradation mechanism and evaluated the enzymatic properties. Then, the chitinase production efficiency was also improved by optimizing the host strain and signal peptide. It was the first time that the chitinase was found in *B. paralicheniformis*, and this chitinase had great application potential for the degradation of chitin.

## 2. Materials and Methods

### 2.1. Chemicals

The restriction endonuclease and T4 DNA ligase used in this study were purchased from TransGen Biotech Co., Ltd. (Beijing, China). The One-Step PAGE Gel Fast Preparation Kit (12%) and two Taq Master Mixes were provided by Vazyme Biotech Co., Ltd. (Nanjing, China). The chitin powder was acquired from Aladdin Biochemical Technology Co., Ltd. (Shanghai, China). The tryptone and yeast powder were acquired from Lanjeco Technology Co., Ltd. (Beijing, China). The DNS reagents were provided by Yuanye Biotechnology Co., Ltd. (Shanghai, China). The rest of the chemical reagents were purchased from Sinopharm Group Chemical Reagent Co., Ltd. (Shanghai, China).

### 2.2. Culture Medium

The Luria-Bertani Broth liquid medium contained NaCl (10.0 g/L), tryptone (10.0 g/L), and yeast extract (5.0 g/L). The composition of the colloidal chitin medium included K_2_HPO_4_ (0.7 g/L), KH_2_PO_4_ (0.3 g/L), MgSO_4_·7H_2_O (0.5 g/L), FeSO_4_·7H_2_O (0.01 g/L), ZnSO_4_ (0.001 g/L), and 1% colloidal chitin powder (*w*/*v*). To obtain the colloidal chitin agar medium, 1.5% agar powder (*w*/*v*) was added to the colloidal chitin medium. The chitinase fermentation medium included tryptone (80.0 g/L), yeast powder (25.0 g/L), NH_4_Cl (6.0 g/L), and K_2_HPO_4_·7H_2_O (6.5 g/L). Chitin degradation medium was added with 1% colloidal chitin powder (*w*/*v*) to the chitinase fermentation medium.

### 2.3. Colloidal Chitin Preparation

The powdered chitin was mixed with 85% phosphoric acid in a 1:10 (*w*/*v*) ratio and incubated at 37 °C for 12 h. Subsequently, 1 mol/L sodium dihydrogen phosphate and 1 mol/L disodium hydrogen phosphate were added to the mixture in a ratio of 1:1:2 (*v*/*v*/*v*). Then, the reactant was centrifuged at 10,000× *g* for 10 min, and the precipitate was freeze-dried to obtain chitin colloids.

### 2.4. Screening and Identification of Wild Bacteria

The soil samples were mixed with sterile water in a 1:9 (*w*/*v*) ratio, incubated at 80 °C for 10 min; then, the suspension was centrifuged at 10,000× *g* for 10 min. A total of 1 mL supernatant was taken and spread on colloidal chitin agar. After culture at 37 °C for 3 days, colonies with transparent rings were transferred into chitin culture medium and inoculated at 140 rpm for 3 days. After centrifugation at 10,000× *g* for 10 min, the supernatant was sterilized through a 0.22 μm filter membrane and added to the wells of the colloidal chitin agar plates for incubation at 37 °C. The strain with the largest transparent circle diameter was selected. The sequences of screened strains were amplified using 16S rDNA primers (27F: AGAGTTTGATCCTGGCTCAG, 1492R: GGTTACCTTGTTACGACTT) and sequenced by Tsingke Biotechnology Co., Ltd (Beijing, China). The sequence similarity analysis was performed by BLAST, and a phylogenetic tree was generated using MEGA 11.

### 2.5. Construction of Recombinant Strains

The construction of the recombinant strains followed the procedure reported in our previous study [39]. Table 1 lists all strains and plasmids and Table 2 lists all designed primers. The *ch1* gene fragment was acquired from *B. paralicheniformis* HL37; then, restriction enzymes *Xba*I and *Bam*HI were used to digest the *ch1* gene fragment and pT17 plasmid. Subsequently, T4 DNA ligase was utilized to ligate the *ch1* gene fragment and pT17 plasmid to generate the expression plasmid pT17-*ch1*. Finally, the pT17-*ch1* was electro-transformed into *B. amyloliquefaciens* HZ12 to obtain the recombinant strain HZ12/pT17-*ch1*. All recombinant strains in this study were constructed by using the same procedure. 

### 2.6. Shake Flask Fermentation

The single colony of recombinant strain was selected and inoculated into 5 mL LB broth at 37 °C at 180 rpm for 12 h to obtain the seed solution. Then, a total of 1.5 mL seed solution was added into 50 mL chitinase fermentation medium and incubated at 37 °C at 180 rpm for 48 h. Subsequently, the fermentation broth was transferred into a 2 mL centrifuge tube and centrifugated at 10,000× *g* for 5 min to obtain the crude enzyme solution. 

### 2.7. Determination of Chitinase Activity

A total of 500 μL fermentation supernatant and 500 μL 1% colloidal chitin (*w*/*v*) was filled with tube A, and, to tube B (blank control), 500 μL fermentation supernatant and 500 μL distilled water were added. Then, tubes A and B were incubated at 55 °C for 30 min, respectively. After inactivation at 100 °C for 5 min, tubes A and B were centrifugated at 10,000× *g* for 5 min. Then, the 500 mL supernatant and 500 mL DNS reagent were transferred into colorimetric tubes, respectively. The reactants were heated at 100 °C for 5 min and cooled to room temperature. Finally, 4 mL distilled water was added to colorimetric tubes and the absorbance was measured at a wavelength of 540 nm.

(1) $$X=m\cdot A\cdot n\cdot \frac{1000}{221.208}\cdot (t\cdot B\cdot C)$$

*m*: The relative molecular mass of GlcNAc;

*A*: The final volume of the reaction solution in the experimental group;

*n*: Sample dilution ratio;

*t*: Reaction time;

*B*: The volume of the supernatant after incubation;

*C*: The volume of the supernatant from the fermentation broth.

### 2.8. SDS-PAGE Analysis

Trichloroacetic acid (TCA) was added to precipitate protein in fermentation supernatants [40]. A solution of TCA was combined with the fermentation supernatant at a ratio of 1:9 (*v*/*v*) and refrigerated at 4 °C for 12 h. After centrifugation at 10,000× *g* for 10 min, the solid precipitate was rinsed three times with 200 mL ethanol and then placed at 37 °C for 5 min. Subsequently, 15 μL 8 mmol/L urea, 15 μL 2 mmol/L thiourea, and 15 μL 2 × SDS buffer were sequentially added to solid precipitate. The mixture was placed in boiling water at 100 °C for 5 min. Subsequently, 10 μL of the mixture and 180 kDa pre-stained protein marker were added to different wells of the SDS-PAGE gel for electrophoretic analysis.

### 2.9. Analysis of GlcNAc and (GlcNAc)_2_ by HPLC

The HPLC detection method for GlcNAc and (GlcNAc)_2_ refers to previous research [41]. Firstly, the fermentation broth was centrifuged at 10,000× *g* for 10 min. Then, the supernatant was diluted 40 times and filtered through 0.22 μm filter membrane. The GlcNAc and (GlcNAc)_2_ were analyzed using an Agilent 1260 HPLC equipped with Bio-Rad Aminex HPX-87H was purchased from Bio-Rad Laboratories (San Clara, CA, USA). The column temperature was controlled at 40 °C and the injection volume was 10 μL. The mobile phase was 5 mmol/L aqueous sulfuric acid and the flow rate was 0.6 mL/min. 

### 2.10. Molecular Docking Simulation

The 3D structure of chitinase CH1 in the *B. paralicheniformis* HL37 was predicted through AlphaFold2 [42], which was provided by Shenzhen Beikunyun Supercomputing (https://www.bkunyun.com, accessed on 2 March 2024). The colloidal chitin fragment (GlcNAc)_3_ was obtained through PumChem (https://pubchem.ncbi.nlm.nih.gov, accessed on 2 March 2024). Furthermore, the molecular docking in this study was simulated by Discovery Studio 2019 [43], and the energy minimization was performed before docking. Additionally, the visual analysis of proteins was through PyMOL 2.5 software.

### 2.11. Statistical Analysis

Each group was conducted by using three parallel experiments. The statistical analysis was calculated by SPSS 20.0. The software program Origin 8.5 was used to make the graphs.

## 3. Results and Discussion

### 3.1. Screening and Identification of Chitin-Degrading Bacteria

**Figure 1:**
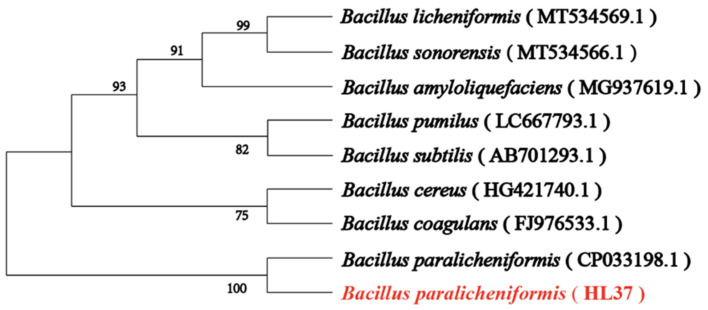
The 16S rDNA phylogenetic tree constructed using MEGA 11 software. Numbers in parentheses indicate the sequence accession numbers of the representative organisms.

In order to screen for thermostable strains, the wild bacteria cultures were placed in an 80 °C water bath for 10 min. Then, the transparent circle method was used to screen the target strain, and a thermostability strain that could efficiently degrade chitin was successfully obtained, named HL37. Then, the 16S rDNA fragment of HL37 was amplified by PCR using 16S rDNA primers and compared by BLAST, which showed that HL37 had the highest similarity to *B. paralicheniformis* (CP033198.1). To further determine the evolutionary relationship of HL37, some representative *Bacillus* genera were selected to construct a phylogenetic tree. As shown in Figure 1, HL37 had a higher affinity with *B. paralicheniformis* compared to other *Bacillus*, further confirming that HL37 belonged to *B. paralicheniformis*.

Currently, several studies showed that *Bacillus* had the ability to degrade chitin, including *Bacillus aryabhattai*, *Bacillus licheniformis*, *Bacillus velezensis*, and *B. subtilis* [29,31,44,45]. In addition, Iqbal et al. predicted the potential chitinase existing in *B. paralicheniformis* through genomic sequencing and annotation, while there was no experimental evidence to support the conclusion [46]. In this study, it was the first time discovering that *B. paralicheniformis* HL37 had the ability to degrade chitin. 

### 3.2. Identification of Chitinase Genes

**Figure 2:**
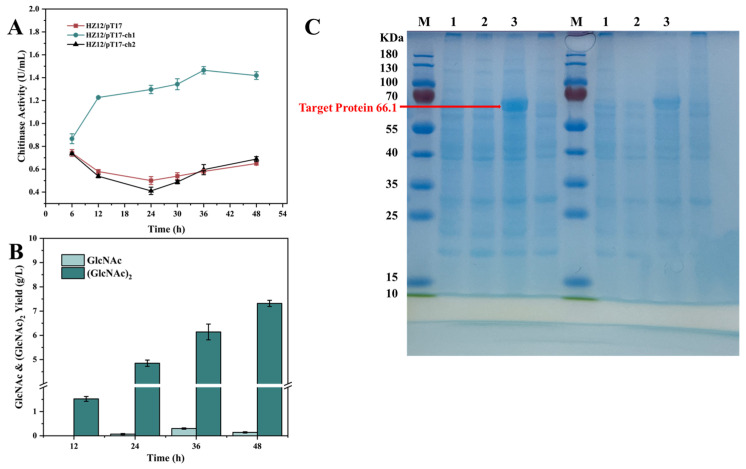
Identification of the chitinase. (**A**) The recombination chitinase activity at different culture times in HZ12/pT17, HZ12/pT17-*ch1*, and HZ12/pT17-*ch2*. (**B**) The yield of GlcNAc and (GlcNAc)_2_ produced by degradation of colloidal chitin with HZ12/pT17-*ch1*. (**C**) SDS-PAGE analysis of recombinant chitinase CH1. Lane M was a molecular weight marker and lane 1 to lane 3 represented HZ12, HZ12/pT17, and HZ12/pT17-*ch1*, resepectively.

In order to discover the key chitinase in *B. paralicheniformis* HL37, we screened two genes *ch1* and *ch2* from *B. paralicheniformis* on KEGG and constructed the recombinant expression strains HZ12/pT17-*ch1* and HZ12/pT17-*ch2*. As shown in Figure 2A, there was no significant difference in chitinase activity between the recombinant strain HZ12/pT17-*ch2* and the control strain HZ12/pT17, and the chitinase activity of the HZ12/pT17-*ch1* was much higher than that of the control strain. When the fermentation time reached 36 h, the chitinase activity of HZ12/pT17-*ch1* reached 1.46 U/mL, which increased by 118% compared to control strain HZ12/pT17. This result indicated that *ch1* was the key gene for chitin degradation in strain *B. paralicheniformis* HL37.

In addition, HZ12/pT17-*ch1* could hydrolyze colloidal chitin to produce GlcNAc and (GlcNAc)_2_ (Figure 2B), and it could obtain 0.14 g/L GlcNAc and 7.3 g/L (GlcNAc)_2_, respectively. A previous study found that GlcNAc and (GlcNAc)_2_ had significant anti-inflammatory and antitumor effects [47]. Especially, (GlcNAc)_2_ showed the function of relieving type 2 diabetes [32], which had the potential to be developed into a health food. Therefore, the *ch1* gene encoded chitinase provided potential resources for the development of health foods. Furthermore, the gene *ch1* had similar functions with gene *rch1* in *Bacillus clausii* TCCC 11004; it could hydrolyze colloidal chitin to produce GlcNAc and (GlcNAc)_2_ [48]. Then, the *ch1* gene encoding chitinase CH1 was characterized by SDS-PAGE (Figure 2C). The molecular weight of CH1 was 66.1 KDa, which was consistent with the predicted target protein. In addition, the isoelectric point of CH1 was 5.14, which indicated CH1 was a stable hydrophilic protein. These results were similar with the chitinase RCH1 (66.7 KDa) from *B. clausii*, and the isoelectric point of RCH1 was 4.51 [48]. 

### 3.3. Bioinformatics Analysis of CH1

**Figure 3:**
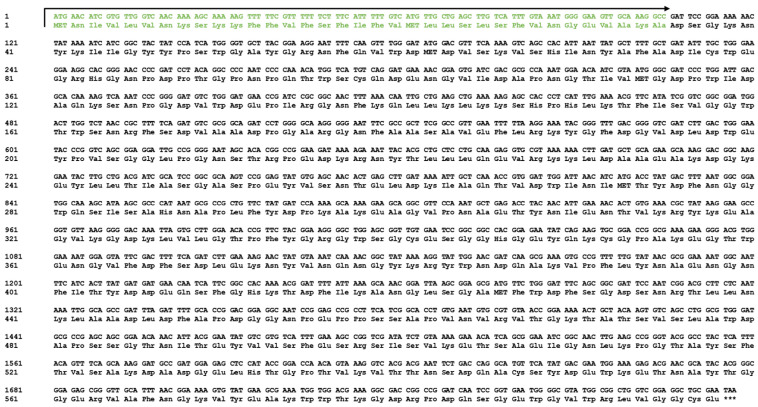
The base sequence and deduced amino acid sequence of the *ch1* gene. The signal peptide sequence is highlighted in green.

**Figure 4:**
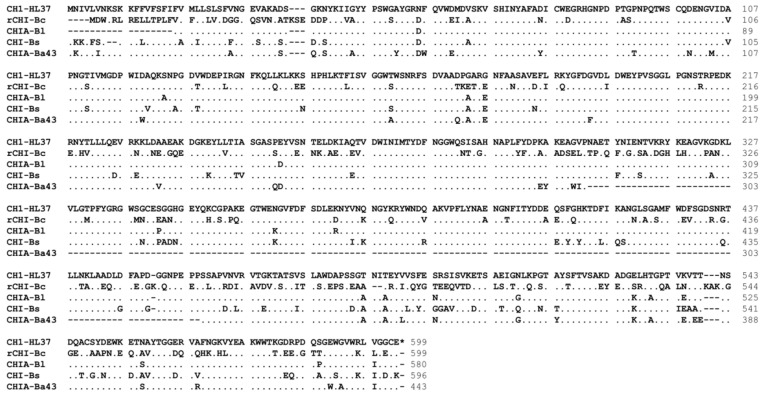
Comparative analysis of amino acid sequences between CH1 (HL37) and four chitinases, namely rCHI-Bc, CHIA-Bl, CHI-Bs, and CHIA-Ba43.

In order to further investigate the sequence composition of CH1 protein, we analyzed the amino acid sequence using SignalP 6.0. As shown in Figure 3, the result indicated that CH1 contained 599 amino acids and the signal peptide was distributed between 1 and 35 amino acids. Among them, amino acids 8–11 were a strongly hydrophilic sequence formed by Lys-Ser-Lys-Lys, while the retaining amino acids were strongly hydrophobic sequences ending with Ala-Lys-Ala. Subsequently, we selected several homologous proteins for sequence alignment to further determine their specific functional sites. According to CAZy-Home database prediction, chitinase CH1 was classified into Glycoside Hydrolase Family 18 and Glycoside Hydrolase Family 19, which possessed enzymatic properties of these two families. As shown in Figure 4, our CH1 protein (CH1-HL37) exhibited 63.9% similarity to rCHI-Bc obtained from *B. clausii* (MW250867.1) [48], 96.9% similarity to CHIA-Bl obtained from *B. licheniformis* (FJ465148.1) [49], 85.0% similarity to CHI-Bs obtained from *B. subtilis* (AF069131.1) [31], and 88.3% similarity to CHIA-Ba43 obtained from *Bacillus altitudinis* (MT331611.1) [50]. The high homologous sequence also indicated that CH1-HL37 possessed chitinase properties.

### 3.4. Analysis of the Mechanism of Action of Chitinase

**Figure 5:**
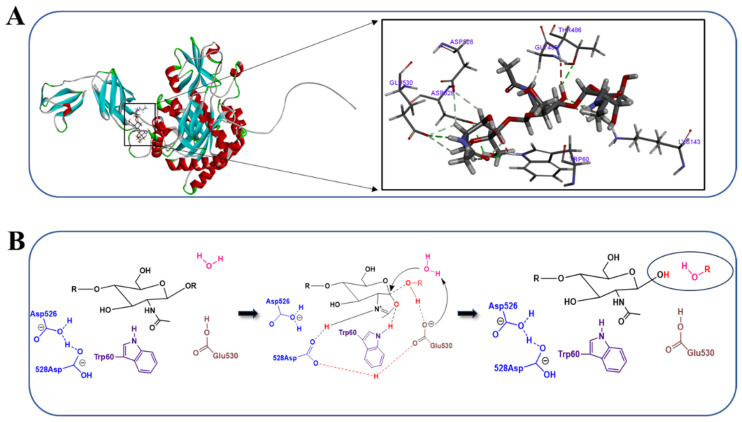
The catalytic function of chitinase CH1. (**A**) The protein structure of CH1 and its molecular docking with (GlcNAc)_3_. (**B**) Schematic diagram of the catalytic mechanism of chitinase CH1.

To further elucidate the catalytic mechanism of chitinase CH1, we used AlphaFold2 to predict the protein structure (Figure 5A), and the results indicated that chitinase CH1 belonged to the GH18 family, which had an eight-strand α/β barrel-shaped structure [51]. Then, we obtained the structure of colloidal chitin fragments (GlcNAc)_3_ from Pumchem, and Discovery Studio 2019 software was used to simulate molecular docking between chitinase CH1 and (GlcNAc)_3_. As shown in Figure 5A, some amino acids in chitinase played an important catalytic role, including Trp60, Lys143, Gly485, and Thr486, and these amino acids were responsible for anchoring substrates during the catalytic process. Then, the remaining amino acids Asp526, Asp528, and Glu530 were conserved catalytic triplets and active centers in chitinase.

Furthermore, we analyzed the specific catalytic mechanism of CH1 (Figure 5B). When the substrate (GlcNAc)_3_ entered the active center of the enzyme, the structure of substrate changed from a chair conformation to a boat conformation under the action of amino acid residues. Then, it caused the carboxyl oxygen atom of the N-acetyl group to approach the heterocyclic carbon (C1), which initiated a nucleophilic attack. Subsequently, Trp60 formed a hydrogen bond with the carboxyl oxygen atom to stabilize the structure, while Asp528 flipped and formed a hydrogen bond with the N-acetyl group to stabilize the intermediate structure. After Asp528 flipped, it approached Glu530 and formed hydrogen bonds to activate proton donors. Then, it would break glycosidic bonds to form intermediate oxazoline ions. Subsequently, the complex of Asp528- and Glu530-catalyzed water molecules aroused nucleophilic attacks on C1 and the intermediate of oxazoline ions disintegrated. At the same time, the Glu530 returned to its initial state and the Asp528 flipped back to its initial position with the assistance of Asp526. Finally, the product was released and hydrolysis was completed [52].

### 3.5. The Enzymatic Properties of Chitinase CH1

**Figure 6:**
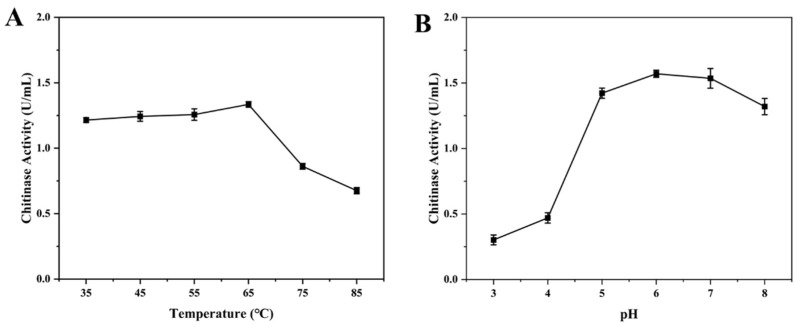
Effects of different reaction conditions on the chitinase catalytic activity. (**A**) Effects of different reaction temperatures on the chitinase catalytic activity. (**B**) Effects of different reaction pH on the chitinase catalytic activity.

**Figure 7:**
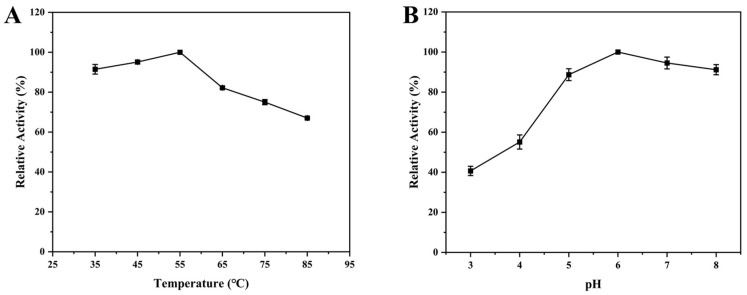
The residual chitinase activities after exposure to different conditions for 30 min. (**A**) The residual chitinase activities after exposure to different temperatures for 30 min. (**B**) The residual chitinase activities after exposure to different pH for 30 min.

To further understand the properties of chitinase in detail, we investigated the performance of chitinase at different temperatures and pH. As shown in Figure 6A, the CH1 protein maintained high chitinase activity in the range of 35~65 °C. After exceeding 65 °C, the chitinase activity significantly decreased and the maximum activity occurred at 65 °C. In addition, we found that the CH1 protein retained high chitinase activity in the pH range of 5~8 (Figure 6B). The chitinase activity significantly decreased below pH 5, and the activity reached maximum when pH was 6. Similarly, Yuan et al. also reported that the optimal pH of chitinase from *B. subtilis* was 5 and the optimal temperature was 60 °C [53]. Subsequently, we further investigated the tolerance of chitinase to temperature and pH. When the temperature increased, there was no significant change in enzyme activity below 55 °C (Figure 7A). The enzyme activity began to decrease when the temperature exceeded 65 °C and it still maintained over 60% activity in the range of 65~85 °C, indicating that the chitinase CH1 had good thermostability. In addition, the chitinase activity remained above 80% in the pH range of 5~8 and the chitinase activity significantly decreased when pH was below 5 (Figure 7B). A study found that the chitinase from *Trichoderma harzianum* only maintained 35.8% activity at 50 °C [28]. In this study, the chitinase CH1 had good thermostability, which contributed to the commercial application.

### 3.6. Effects of Different Host Bacteria and Signal Peptides on Chitinase Activity

**Figure 8:**
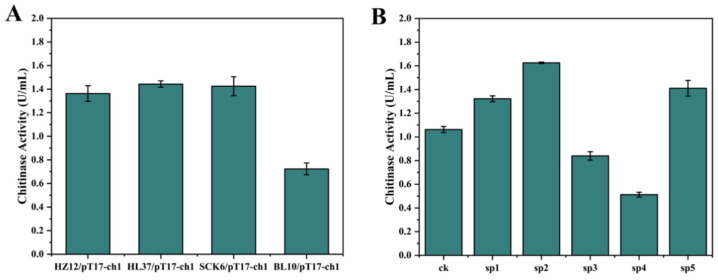
Optimization of the expression of chitinase CH1. (**A**) The effect of different host bacteria on chitinase CH1 activity. (**B**) The effect of different signal peptides on chitinase CH1 activity.

Different host strains might have different impacts on the expression of proteins [54]. To further enhance the fermentation activity of chitinase CH1, we screened the chassis strains for expressing chitinase. The recombinant vector pT17-*ch1* was transferred into *B. paralicheniformis* HL37, *B. subtilis* SCK6 [55], and *B. licheniformis* BL10 [56], respectively, generating engineering strains HL37/pT17-*ch1*, BL10/pT17-*ch1*, and SCK6/pT17-*ch1*. Then, we measured the chitinase activity of four strains (including HZ12/pT17-*ch1*) after fermentation. As shown in Figure 8A, the recombinant chitinase CH1 indicated the lowest activity for BL10/pT17-*ch1* and the remaining three recombinant strains HZ12/pT17-*ch1*, HL37/pT17-*ch1*, and SCK6/pT17-*ch1* showed no significant difference. Therefore, *B. amyloliquefaciens* HZ12 was chosen as the optimal host strain.

The type of signal peptide could significantly affect the secretion level of recombinant proteins [57]. Therefore, we used SignalP 6.0-DTU Health Tech-Bioinformatic Services to screen and analyze the signal peptide library. Then, five signal peptides were selected for expression of chitinase, namely sp1, sp2, sp3, sp4, and sp5, which had 35, 35, 17, 29, and 27 amino acids, respectively. Subsequently, we used SOE-PCR (gene splicing by overlap extension PCR) to connect signal peptides with gene *ch1* and constructed five vectors, namely pT17-sp1*ch1*, pT17-sp2*ch1*, pT17-sp3*ch1*, pT17-sp4*ch1*, and pT17-sp5*ch1*, respectively. These vectors were electrotransformed into HZ12 competent cells and obtained five engineering strains, namely HZ12/pT17-sp1*ch1*, HZ12/pT17-sp2*ch1*, HZ12/pT17-sp3*ch1*, HZ12/pT17-sp4*ch1*, and HZ12/pT17-sp5*ch1*, respectively. As shown in Figure 8B, chitinase activities of three engineering strains were higher than CK (HZ12/pT17-*ch1*), including HZ12/pT17-sp1*ch1*, HZ12/pT17-sp2*ch1*, and HZ12/pT17-sp5*ch1*, respectively. Among them, the HZ12/pT17-sp2*ch1* had the highest enzyme activity, which reached 1.73 U/mL, and it was 63.0% higher than that in CK. This indicated that the signal peptide sp2 was effective to enhance the expression level of recombinant chitinase CH1.

## 4. Conclusions

In summary, this study successfully screened a strain HL37 that efficiently degraded chitin and identified its key chitinase gene *ch1* through recombinant expression. Moreover, the molecular weight, amino acid sequence, and protein structure of chitinase CH1 were analyzed, and the catalytic mechanism was also explained. Furthermore, the enzymatic properties of chitinase were investigated. The optimal reaction temperature was 65 °C and the optimal pH was 5.0. Through screening of host strains and signal peptides, the enzyme activity of chitinase CH1 was further enhanced. Therefore, the chitinase discovered in this study had potential significance for industrial production.

## Figures and Tables

**Table 1:** Strains and plasmids used in this study.

Strains or Plasmids	Characteristics	Source
Strains		
*E. coli* DH5α	F− Φ80d/lacZΔM15, Δ(lacZYAargF) U169, recA1, endA1, hsdR17 (rK −, mK +), phoA, supE44, λ−, thi-1, gyrA96, relA1	stored in lab
*B. paralicheniformis* HL37	wild type	this study
*B. amyloliquefaciens* HZ12	wild type	stored in lab
*B. subtilis* SCK6	Erm^R^,1A751 derivate, *lacA*::P*_xylA_-comK*	stored in lab
*B. licheniformis* BL10	WX-02 (Δ*hag*; Δ*mpr*; Δ*vpr*; Δ*aprX*; Δ*epr*; Δ*bpr*; Δ*wprA*; Δ*aprE*; Δ*amyL*; Δ*bprA*)	stored in lab
HZ12/pT17	HZ12 harboring the plasmid pT17	stored in lab
HZ12/pT17-*ch1*	HZ12 harboring the plasmid pT17-*ch1*	this study
HZ12/pT17-*ch2*	HZ12 harboring the plasmid pT17-*ch2*	this study
HL37/pT17-*ch1*	HL37 harboring the plasmid pT17-*ch1*	this study
BL10/pT17-*ch1*	BL10 harboring the plasmid pT17-*ch1*	this study
SCK6/pT17-*ch1*	SCK6 harboring the plasmid pT17-*ch1*	this study
HZ12/pT17-sp1*ch1*	HZ12 harboring the plasmid pT17-sp1*ch1*	this study
HZ12/pT17-sp2*ch1*	HZ12 harboring the plasmid pT17-sp2*ch1*	this study
HZ12/pT17-sp3*ch1*	HZ12 harboring the plasmid pT17-sp3*ch1*	this study
HZ12/pT17-sp4*ch1*	HZ12 harboring the plasmid pT17-sp4*ch1*	this study
HZ12/pT17-sp5*ch1*	HZ12 harboring the plasmid pT17-sp5*ch1*	this study
Plasmids		
pT17	pHY300PLK + p43 + TamyL	stored in lab
pT17-*ch1*	pHY300PLK + p43 + TamyL + *ch1* from *B. paralicheniformis* HL37	this study
pT17-*ch2*	pHY300PLK + p43 + TamyL + *ch2* from *B. paralicheniformis* HL37	this study
pT17-sp1*ch1*	pT17-*ch1* + sp1 from *B. paralicheniformis* chitinase gene	this study
pT17-sp2*ch1*	pT17-*ch1* + sp2 from *B. altitudinis* chitinase gene	this study
pT17-sp3*ch1*	pT17-*ch1* + sp3 from *B. licheniformis* chitinase gene	this study
pT17-sp4*ch1*	pT17-*ch1* + sp4 from *Apre* gene	this study
pT17-sp5*ch1*	pT17-*ch1* + sp5 from *Npre* gene	this study

**Table 2:** Primers used in this study.

Primer Name	Sequence of Primer (5′ to 3′)
pT17-F	GCGGAGCCTATGGAAAAAC
pT17-R	TGGGAGAGAGTTCAAAATTGATCC
*ch*1-F	GC**TCTAGA**ATGTTGCTGAGCTTGTCATTT
*ch1*-R	CG**GGATCC**TTATTCGCAGCCTCCGA
*ch2*-F	GC**TCTAGA**ATGAAGATAGCCGCTTCATC
*ch2*-R	CG**GGATCC**TTACTTCACATTAAGCCTGTACTTT
sp1*ch1*-AF	GC**TCTAGA**ATGAACATCGTGTTGGTCAAC
sp1*ch1*-AR	ATTTTATAGTTTTTTCCGGAATCGGCCTTTGCAACTTCCC
sp1*ch1*-BF	GGGAAGTTGCAAAGGCCGATTCCGGAAAAAACTATAAAAT
sp1*ch1*-BR	CG**GGATCC**TTATTCGCAGCCTCCGA
sp2*ch1*-AF	GC**TCTAGA**ATGAAAATCGTGTTGATCAACA
sp2*ch1*-AR	ATTTTATAGTTTTTTCCGGAATCGGCTTTTGCAACTTCCCC
sp2*ch1*-BF	GGGGAAGTTGCAAAAGCCGATTCCGGAAAAAACTATAAAAT
sp2*ch1*-BR	CG**GGATCC**TTATTCGCAGCCTCCGA
sp3*ch1*-AF	GC**TCTAGA**TTTGTCATGTTGCTGAGCTT
sp3*ch1*-AR	ATTTTATAGTTTTTTCCGGAATCGGCTTTTGCAACTTCCC
sp3*ch1*-BF	GGGAAGTTGCAAAAGCCGATTCCGGAAAAAACTATAAAAT
sp3*ch1*-BR	CG**GGATCC**TTATTCGCAGCCTCCGA
sp4*ch1*-AF	GC**TCTAGA**ATGAGAAGCAAAAAATTGTGG
sp4*ch1*-AR	ATTTTATAGTTTTTTCCGGAATCAGCCTGCGCAGACATGT
sp4*ch1*-BF	ACATGTCTGCGCAGGCTGATTCCGGAAAAAACTATAAAAT
sp4*ch1*-BR	CG**GGATCC**TTATTCGCAGCCTCCGA
sp5*ch1*-AF	GC**TCTAGA**GTGGGTTTAGGTAAGAAATTGTC
sp5*ch1*-AR	AAATGACAAGCTCAGCAACATAGCCTGAACACCTGGCAG
sp5*ch1*-BF	CTGCCAGGTGTTCAGGCTATGTTGCTGAGCTTGTCATTT
sp5*ch1*-BR	CG**GGATCC**TTATTCGCAGCCTCCGA

Note: restriction sites highlighted in bold.

## Data Availability

The original contributions presented in the study are included in the article, further inquiries can be directed to the corresponding author.
